# Phosphopeptide Neoantigens as Emerging Targets in Cancer Immunotherapy

**DOI:** 10.33696/cancerimmunol.6.094

**Published:** 2024

**Authors:** Tyagi Apoorvi, Patskovsky Yury, Voloshyna Iryna, Krogsgaard Michelle

**Affiliations:** 1 Department of Pathology, NYU Grossman School of Medicine, New York, NY, USA; 2 Laura and Isaac Perlmutter Cancer Center at NYU Langone Health, New York, NY, USA

**Keywords:** Post-translational modifications, Immunopeptidome, Phosphorylation, Neoantigens, Immune checkpoint blockade therapies

## Abstract

Protein post-translational modifications play a vital role in various cellular events essential for maintaining cellular physiology and homeostasis. In cancer cells, aberrant post-translational modifications such as glycosylation, acetylation, and phosphorylation on proteins can result in the generation of antigenic peptide variants presented in complex with MHC molecules. These modified peptides add to the class of tumorspecific antigens and offer promising avenues for targeted anti- cancer therapies. In this review, we focus on the role of phosphorylated peptides (p-peptides) in cancer immunity. We discuss the mechanisms by which the phosphorylated moiety modifies the structural features and binding properties of p-peptides with MHC, compared to their non-phosphorylated counterparts. Additionally, we review recent work on how the HLA-B*07-specific p-peptide, pMLL_747–755_, interacts with its cognate TCR. Altogether, p-peptides are emerging as a novel class of tumor-specific antigens, expanding the range of targets in cancer immunotherapy.

## Introduction

Immune checkpoint blockade (ICB) therapies have revolutionized the treatment landscape for various malignancies [1–5]. Clinical benefits from ICB in patients with advanced cancers are directly associated with high tumor mutational burden (TMB) [6,7], as tumors with elevated TMB, such as melanoma, tend to generate highly immunogenic tumor-specific antigens known as neoantigens [6,8–10]. Most neoantigens arise from random passenger mutations, with a significant proportion being unique and not commonly shared across patients.

**Figure 1.**
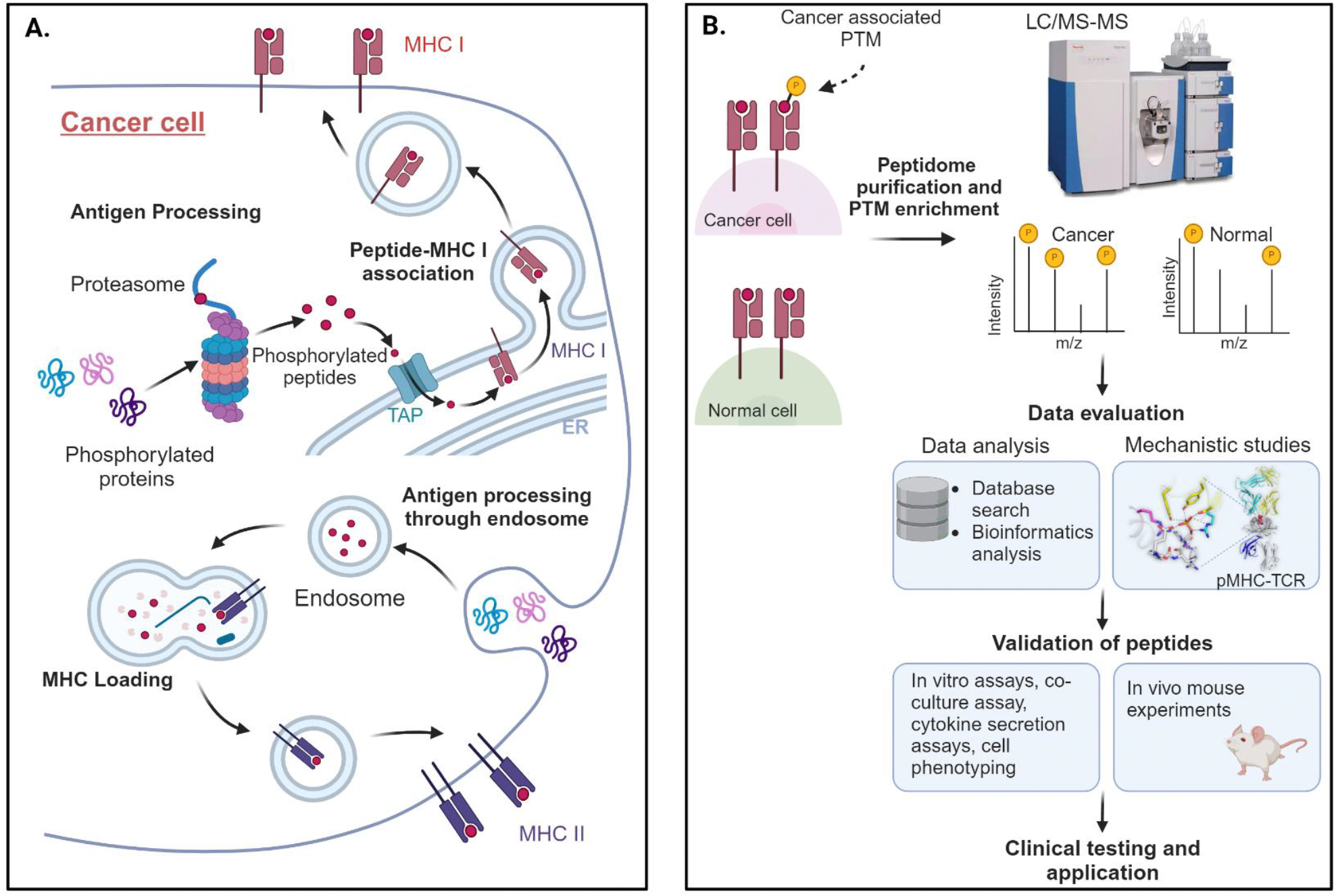
Phosphorylated antigen generation and their utilization for antigen-based treatment workflow in cancers. (**A**) Phosphorylated proteins are intracellularly processed and presented in complex with MHC I or MHC II molecules on cell surface. (**B**) The immunopeptidome purification and enrichment from cancer and normal tissues is done and peptide sequences are tested by Liquid Chromatography with tandem mass spectrometry (LC/MS-MS). The phosphorylated peptides from the immunopeptidome are evaluated using in silico tools for data mining and mechanistic studies and validated by both, *in vitro* and *in vivo* assays before selection for cancer vaccines. Created with BioRender.com.

Aside from mutations, neoantigens can also originate from aberrant post-translational modifications, including glycosylation, O-linked β-N-acetylglucosamine (O-GlcNAc), and phosphorylation [11,12], as well as dysregulated RNA splicing [13–15], proteasome processing, and transporter-associated aberrant antigen processing (TAP) [11,12,16,17]. Some of these post-translational modifications are cancer-specific but not patient-specific, making them promising shared tumor antigens and potential therapeutic targets [18]. For example, Dao *et al.* and Engelhard *et al.* highlighted the importance of cancer-specific phosphopeptides (p-peptides), such as those derived from insulin receptor substrate 2 (pIRS2_1097–1105_) and breast cancer antiestrogen resistance 3 (pBCAR3_126–134_), as viable targets for cancer immunotherapy [19,20]. Additionally, O-GlcNAc-modified peptides associated with MHC-I (HLA-B*07:02) were identified as potential neoantigens in leukemia [21], suggesting that despite a low TMB, leukemias could still be highly immunogenic. Various *in vitro*-generated, peptide-specific T cell lines have been shown to specifically recognize post-translationally modified peptides, but not their unmodified counterparts, indicating that these modifications can lead to the generation of cancer-specific antigens and TCRs [12,21,22]. Immunogenic peptides resulting from phosphorylation represent an untapped class of neoantigens that could serve as off-the-shelf targets for neoantigen-based cancer immunotherapies. These peptides can also be explored for their potential as “public” tumor antigens, which could be incorporated into shared posttranslationally modified antigen-based treatment regimens across multiple patients. The possible identification and treatment strategy utilizing post translationally modified peptides is illustrated in Figure 1.

Here, we focus on the emerging role of phosphorylated tumor antigens in enhancing tumor immunogenicity and developing anti-cancer therapies. Phosphorylated antigens offer a diverse landscape, as variations in phosphorylation sites and patterns give rise to a wide array of potential epitopes. This diversity benefits immunotherapy by providing multiple immune targets, which reduces the likelihood of tumor immune escape. Currently, advanced approaches for identifying the cancer phosphopeptidome, leveraging both sensitive analytical and computational tools, are being used. A list of phosphorylation-associated databases and tools is provided in Table 1.

Incorporating artificial intelligence (AI) into these predictive tools could significantly enhance the accuracy of phosphorylation site prediction and peptide selection. AI models, trained using large-scale data from comprehensive databases such as UniProt and PhosphoSitePlus, can predict phosphorylation sites with high accuracy. Tools such as NetPhos which utilizes neural networks, facilitate the prediction of potential phosphorylation sites. By integrating AI predictions with experimental validation, these models can be continuously refined through a feedback loop, improving the predictive accuracy of AI-based models over time.

In this review, we summarize the published data on phosphopeptide (p-peptide) neoantigens as possible targets for cancer immunotherapy, describe the mechanisms underlying their immunogenicity, and evaluate their potential clinical applications. We also discuss the likely advantages of p-peptide-based therapies, along with known obstacles and potential solutions.

## Phosphopeptide Antigens in Cancer Immunotherapy

A variety of post-translational protein modifications, including glycosylation, acetylation, phosphorylation, and methylation, have been described in cancer-specific and associated peptides. Some post-translationally modified cancer-associated antigens that elicit an anti-cancer response are listed in Table 2. An imbalance between phosphorylation and dephosphorylation, facilitated by kinases and phosphatases, leads to dysregulated signal transduction pathways, often resulting in various malignancies, including cancer [34,35]. Reports indicate the presence of p-peptides in complex with Class I and Class II HLAs on multiple cancer types, including blood cancers, melanoma, breast cancer, and colorectal cancer (Table 2).

One primary source of dysregulated phosphorylation states and increased expression of phosphorylated antigens in cancer and virally infected cells is the suppression of PP2A, a critical phosphatase that regulates various signaling pathways involved in cell apoptosis, transformation, and proliferation [36–38]. High levels of endogenous PP2A negative regulators, such as SET proteins, or inhibitors, such as CIP2A, are associated with cancer progression [39]. Inhibition of PP2A leads to inactivation of retinoblastoma protein (pRb) and the tumor suppressor p53, two frequently mutated proteins in several cancers [40,41]. Unlike normal cells, where phosphorylation is brief and reversible, PP2A suppression in cancer cells extends the lifetime of phosphorylated proteins. This prolonged phosphorylation results in phosphorylated residues remaining even after proteasomal degradation and being presented as pMHC [38]. Decreased activity of another phosphatase, protein phosphatase 1 (PP1), is also associated with increased presentation of p-peptides as pMHC [50].

Although p-peptides have been detected in pMHC complexes isolated from both healthy and cancerous tissues, several epitopes have been found only in cancer cells, most prominently in melanoma and leukemias [43,51–53]. Interestingly, these dysregulated pathways generate the same phosphor-epitopes across multiple cancer types. The immunogenic properties of p-peptides make them attractive targets for cancer immunotherapy, offering a broad and tumorspecific antigen repertoire. Dysregulated phosphorylation of proteins is a hallmark of oncogenic transformation, and p-peptides can add a new layer of antigenicity to other cancer-specific epitopes. Therefore, p-peptides presented by cancer cells could provide an immunological signature of the ‘transformed self’.

Several studies have highlighted the potential of p-peptides as targets in anticancer therapies. Lin *et al.* [42] demonstrated that immunizing transgenic mice with an HLA-A2-specific p-peptide derived from tumor necrosis factor receptor-associated protein 1 (TRAP1) delayed tumor growth and extended survival. Similarly, HLA-A2-restricted p-peptides derived from IRS2 (pIRS2_1097–1105_ (RVApSPTSGV)), β catenin (pβ catenin_30–39_ (YLDpSGIHSGA)), and CDC25b (pCDC25b_38–46_ (GLLGpSPVRA)) were recognized by specific CD8^+^ T lymphocytes in ovarian carcinoma (COV413), and melanoma cell lines (DM331, SLM2) [43]. Further supporting the clinical relevance, studies involving high risk melanoma patients demonstrated that HLA-A2-specific p-peptides, such as pIRS2_1097–1105_ and pBCAR3_126–134_ (YLDpSGIHSGA), induced CD8^+^ T cell responses 42% and 17% of patients, respectively, in a clinical trial (NCT01846143) involving the pBCAR3 phosphopeptide-tetanus vaccine. Importantly, no grade 3–4 adverse effects, dose-limiting toxicity, or death occurred, while on study, underscoring the safety and immunogenicity of p-peptide vaccines [20].

Additionally, HLA-I-bound p-peptides have been identified in primary colorectal cancer tumors, liver metastases, and colorectal cancer cell lines [54]. CD8^+^ tumor-infiltrating lymphocytes (TILs) specific to p-peptides pTNS3 (VMIGpSPKKV) and pSELH (RRGpSFEVTL) were detected, and peripheral T cell activation was observed with pIRS2, pTNS3, and pSELH, indicating the potential role for p-peptides in colorectal cancer immunogenicity [54].

Few studies have identified MHC-II p-peptides recognized by CD4^+^ T cells, which are critical for the generation of effective and long-term anti-tumor immunity [42,55]. Human CD4^+^ T cells that specifically recognize an HLA-DR1-restricted phosphorylated melanoma-associated MART1 p-peptide (pMART1_100–114_(APPAYEKLpSAEQ)) were isolated from a cultured melanoma cell line [55]. HLA-DR- associated p-peptides have also been identified on human melanoma and Epstein-Barr virus (EBV)- transformed B lymphoblastoid lines [56]. Interestingly, similar results were reported in a separate study of human MHC class II-restricted p-peptides derived from other melanoma and B lymphoblastoid cell lines [57], suggesting a commonality of p-peptide presentation by MHC class II molecules.

The increased expression of intracellular phosphoproteins in dysregulated signaling pathways supports the malignant characteristics of tumor cells, and p-peptides may provide selective targets for immunotherapy. Studies have documented the differential expression of phosphoproteins in progressive tumors compared to normal and primary tumor sites. Penny *et al.* identified 120 HLA-I p-peptides from colorectal (CRC) cell lines, primary tumors, and liver metastases, assessing tumor-resident immunity against these p-peptides. Primary tumors displayed 3 times more p-peptides than healthy colon tissues, while liver metastases presented a 1.5-fold increase in p-peptides compared to primary site tumors. Interestingly, similar numbers of p-peptides were found in neighboring healthy liver tissues [54].

A comparative analysis of the phosphoproteome of colon cancer using patient-matched primary (SW480) and metastatic (SW620) cell lines revealed significant phosphorylation changes in critical cancer proteins in the metastatic SW620 cell line [58]. Aikio *et al.* studied phoshphorylation changes in RNA-binding proteins (RBPs) during prostate cancer development and identified 207 p-peptides originating from 133 RBPs. Phosphorylation patterns were consistent between benign and local prostate cancer. On contrary, there were significant changes between early to metastatic castration-resistant prostate cancer, with a reduction in phosphorylation in nearly one-third of cases and an increase in two-thirds [59]. Another study by Drake *et al.* identified 18 differentially phosphorylated kinases in clinical metastatic castration-resistant prostate cancer tissues [60]. These findings reveal distinct phosphorylation profiles between primary and metastatic tumors, suggesting that characterizing p-peptides across malignancies and tumor stages may uncover new neoantigens.

Despite these promising results, further research is needed to corroborate these findings, particularly regarding tumor progression, and to provide a comprehensive understanding of p-peptide neoantigens. Many p-peptides are commonly expressed across various cancer types and are recognized by healthy donor T cells, indicating that p-peptides could be safely targeted in a broad range of cancers.

The field of precision immunotherapy is rapidly evolving, with improvements in vaccine delivery methods and the use of combinatory immunotherapy strategies [61]. However, data on the safety and efficacy of post-translational neoantigen-based vaccines remain very limited, although the clinical trials show minimal to no adverse effects [20]. We expect a significant enhancement of antitumor efficacy when anti-cancer vaccines are combined with immune checkpoint inhibitors [62,63]. Further improvements in neoantigen delivery systems, such as self-amplifying mRNA and chemically synthesized minimal mRNA (CmRNA), may further enhance cellular trafficking, target specificity, and immunogenicity compared to *in vitro* transcribed mRNA (IVT-mRNA) [63,64].

## Mechanisms of Phosphopeptide Immunogenicity

Limited mechanistic data have thus far hampered efforts to understand the role of post-translational protein modifications, including phosphorylation, in tumor immunity and to explore their potential applications in cancer immunotherapy. Establishing the molecular basis for p-peptide presentation in complex with MHC and their recognition by cognate TCRs would enable the rational design of immunotherapies targeting p-peptides.

Several studies have indicated that the presence of a phosphate group leads to physicochemical and conformational changes in a p-peptide-MHC complex compared to an unmodified wild-type (WT) peptide-MHC, thereby triggering immune recognition of the former [65–67]. The addition of a phosphate group to any peptide significantly alters its physicochemical properties. For instance, the negatively charged phosphate group (charge -2) is a strong hydrogen bond acceptor, which may support electrostatic interactions between the bound p-peptide and HLA, thereby increasing the stability of the binary complex [66,67].

**Figure 2.**
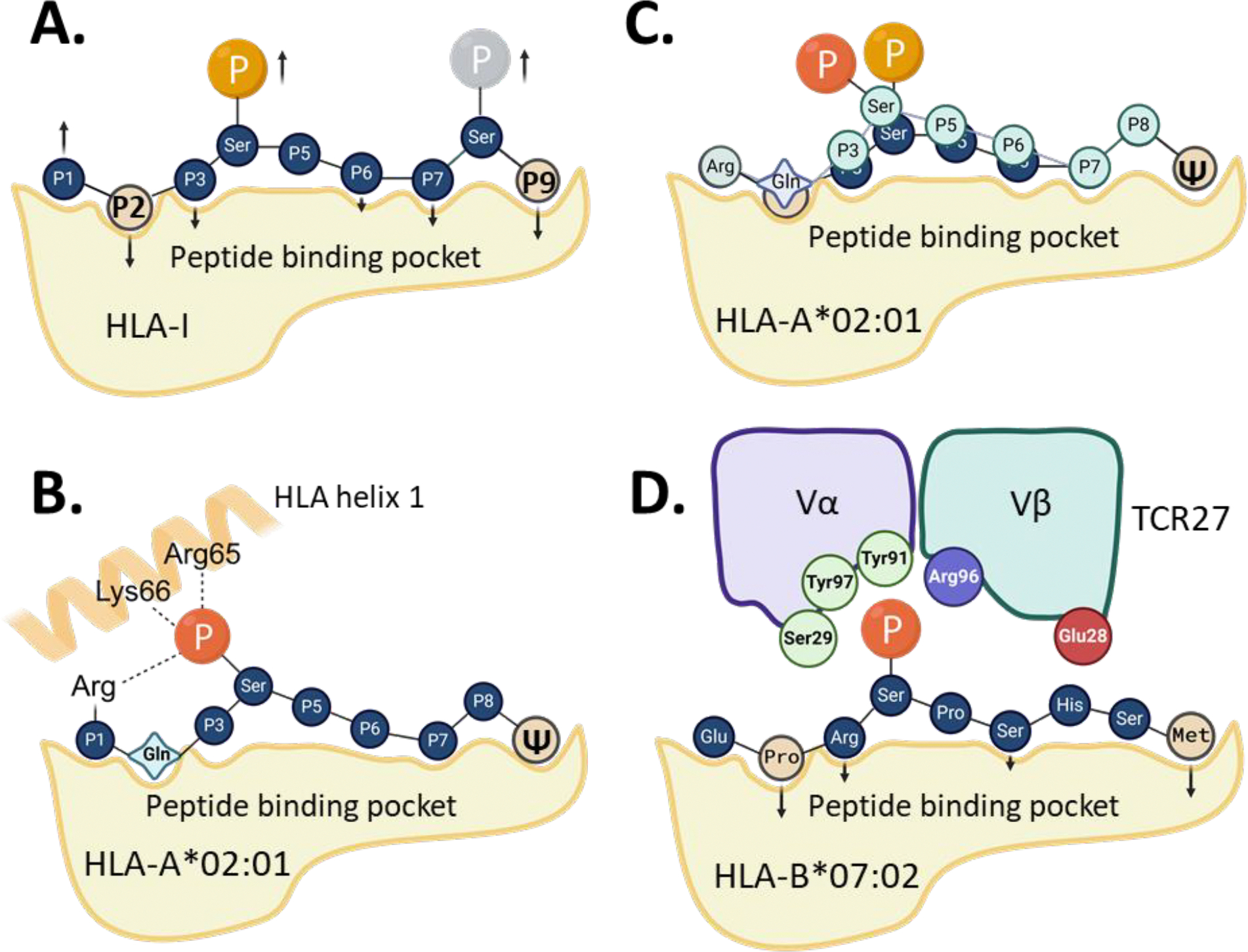
Binding between phosphopeptides and HLA class I molecules. (**A**) The most typical binding pattern between p-peptide and HLA with phosposerine at position P4 and/or P8 of a 9-mer peptide. The arrows show anchor residues (down) or non-anchor residues (up). P2 and P9 are primary anchor residues, others are usually optional and vary between different peptides. (**B**) The binding pattern between p-peptides with a consensus sequence of R/KQx(pS)xxxxΨ and HLA-A*02:01. Hydrogen bonds shown as dotted lines. (**C**) Superimposition between the two typical binding patterns observed for p-peptides in complex with HLA-A*02:01. (**D**) Schematic representation of the interface between HLA-B*07:02, pMLL p-peptide and TCR27 (variable region only). The TCR residues involved in hydrogen bonding with p-peptide are depicted. Ψ - aliphatic amino acid residues. Created with BioRender.com.

In most cases, the anionic nature and sheer size of any phosphorylated residue, such as serine, threonine or tyrosine, likely prevents Class I HLAs from binding a p-peptide with phosphorylated anchor residues. These steric limitations, along with those imposed by kinase-specific phosphorylation patterns [68], result in the majority of HLA Class I p-peptides being phosphorylated at serine in position P4 [69] (Figure 2A). Moreover, many HLA-I-specific p-peptides share a common sequence motif with a positively charged basic arginine or lysine residue at P1 and a proline residue at P5 [52,66,70], reflecting the phosphorylation patterns of 1,4-basophilic (e.g., Akt) and proline-directed protein kinases (e.g., MAP kinases and CDKs) [68].

As a consequence of these restrictions, the phosphoserine residue in a typical p-peptide-HLA complex is a non-anchor residue. It remains at least partially exposed to solvent, making it a primary target for TCRs and a major determinant of p-peptide immunogenicity. The only exception to this rule has been reported for HLA-B*40:02-specific p-peptides, whose binding motif includes glutamic or aspartic acid at the anchor position P2, which may be substituted by phosphoserine [71]. However, p-peptides with pSer-P2 are extremely rare, and no crystal structures have been reported so far to confirm this finding.

The mechanistic data available to date, though limited to phosphoserine-containing peptides, demonstrate that the binding affinity between p-peptide and HLA, as well as the conformation of the binary complex, depend on: (1) HLA type and its peptide specificity, (2) the nature of the p-peptide, including its length, sequence, number of phosphate groups, and the positions of phosphorylated residues, and (3) the prevalence of certain protein phosphorylation motifs in cancer cells, which could limit both the number and types of p-peptides presented as pMHC.

In earlier studies by Zarling and co-authors [43,44,53], a substantial number of high-affinity HLA-A*02:01-specific p-peptides were identified with a glutamine residue in position P2. Non- phosphorylated (non-p) peptides with Gln-P2 (or other polar side chains) are usually poor binders to HLA-A*02:01, which typically prefers peptides Leu-P2 [66]. The unexpectedly high affinity and high immunogenicity observed for several Gln-P2-containing p-peptides prompted subsequent structural and mechanistic studies. Mohammed and co-authors determined the crystal structures of HLA-A*02:01 complexed with several p-peptides with various amino acid residues at P2 [66,67], revealing several unusual features of p-peptides recognized by HLA-A*02:01.

For instance, all the p-peptides with the N-terminal consensus sequence of R/KQx(pS) displayed very high binding affinities (with KD values in a low nanomolar range) to HLA-A*02:01. The pMHC crystal structures show that a phosphate group at pSer-P4 maintains electrostatic interactions with basic Arg and Lys side chains (Arg-P1, Arg65 and Lys66), forming an integral part of the p-peptide-HLA interface (Figure 2B) [22,57,67]. By contrast, non-p-peptides with N-terminal sequences of R/KQxS had much lower binding affinities to HLA-A*02:01, with KD values in the micromolar range. Additionally, the conformations of HLA-bound non-p-peptides and the relative orientations of the Arg-P1, Lys65, and Arg66 side chains differed from those observed in a p-peptide-HLA complex [66,67].

An exception to the affinity rule was the pLSP1 (RQA(pS) IELPSMAV) p-peptide, where the binding affinity was similar to that of a WT peptide (RQASIELPSMAV). The main factor was the peptide size (12 amino acid residues), which led to a similar “bulging” ligand conformation observed in both the non-phospho- and p-peptide N termini, including pSer-P4 and Ser-P4 residues, respectively [67]. Notably, the increase in binding affinity following peptide phosphorylation was dependent on the nature of amino acid residue in position P2, as follows: Q ≫ T ≫ V > M. The affinities between non-p-peptides and HLA-A*02:01 followed the reverse pattern: M > V ≫ T > >Q [65, 67].

Studies by Petersen and co-authors [65] further clarified the mechanisms controlling the binding between p-peptides and HLA-A*02:01. The authors solved the crystal structures of HLA-A*02:01 bound to three distinct p-peptide epitopes, alongside their matching non-p-peptides (Table 3), including pβ-catenin_30–39_ (YLD(pS)GIHSGA), pCDC25b_38–46_, (GLLG(pS) PVRA), and pIRS2_1097–1105_ (RVA(pS)PTSGV). They also measured the binding affinities between these ligands and HLA and, notably, determined that phosphorylation had no effect on the binding between most peptides and HLA- A*02:01, except for the pIRS2 p-peptide, where the affinity was slightly increased compared to IRS2 peptide.

These crystallographic studies revealed a potential mechanism behind the observed affinity data. The structures of HLA-A*02:01 bound to pβ-catenin or pCDC25b epitopes were very similar to those crystallized with their non-phosphorylated counterparts. In all three pMHC structures with above p-peptides [65], the phosphate groups were solvent-exposed and displayed signs of increased flexibility, as indicated by the presence of at least two alternating conformations for Ser-P4 in each structure.

The pβ-catenin_30–39_ peptide has Tyr-P1, whereas Leu-P2 and Ala-P10 serve as its anchor residues. Interestingly, phosphorylation of Ser33 is necessary for ubiquitin-mediated proteasomal degradation of β-catenin [74,75]. Since HLA Class I-restricted peptides are processed via the proteasomal pathway for presentation, there appears to be a link between this specific phosphorylation and p-peptide presentation by HLA-A*02:01.

In contrast, in pCDC25b_38–46_ (GLLG(pS)PVRA), the pSer residue is not in position P4 but at P5. In the CDC25b peptide-HLA-A*02:01 structure, Ser-P5 acts as an anchor residue. However, when phosphorylated, Ser-P5 becomes a non-anchor residue, resulting in conformational differences between the p-peptide and its WT counterpart. Although this conformational change does affect the binding affinity between HLA and the ligands, the P5-phosphorylated p-peptides could be immunogenic.

In a complex between HLA-A*02:01 and pIRS2_1097–1105_, the predominant alternative conformation of the phosphate moiety is positioned within hydrogen-bonding distance from Lys66 and Arg65, which likely explains the increased stability of this complex compared to the non-p-peptide-MHC complex [65]. In summary, the strong binding between p-peptides and HLA-A*02:01 depends on multiple factors, as described in the examples above. However, in general, the structure of a p-peptide-HLA-A*02:01 complex is always different from that of its non-phosphorylated counterpart. This structural difference could potentially prevent cross-recognition between p-peptides and non-p-peptides by the same TCRs.

Binding between p-peptides and HLA-B molecules share certain features similar to those described above for HLA-A*02:01. However, no p-peptide has been reported yet for HLA-B alleles with a binding affinity significantly greater than that of its corresponding non-p-peptide. For example, in phospho-immunopeptidomics studies of B*07:02, B*27:01, B*39:01, and B*40:02 restricted p-peptides (8– 13mers), phosphorylation was observed at position P4 in more than 60% of all peptide ligands [72]. The majority of p-peptides, except those specific to B*39:01, also had a basic arginine at P1 [72]. The HLA-peptide binding motifs were the same for both p-peptides and non-p-peptides, and the presence or absence of Arg/Lys at P1 or pSer at P4 had no significant effect on the binding affinity between p- peptides and HLA-B molecules [22,72].

To determine a molecular basis for these observations, Alpizar and co-authors solved the crystal structure of HLA-B*40:02 in complex with the p-peptide pINCENP_47–55_ (pREF(pS)KEPEL) [72]. The structure revealed pSer-P4 as a non-anchor and solvent-accessible residue with a phosphate moiety pointing out of the binding pocket, making it accessible for interaction with the TCR. No significant conformational differences were observed between the HLA-B*40:02 structures in complex with pINCENP or the non-phosphorylated INCENP peptide. Moreover, contrary to what is observed with HLA-A*02:01, the P1 arginine did not interact with pSer-P4 and had no effect on the conformation of pSer-P4 [72].

Recent data reported by Zhao and co-authors [73] described the crystal structures of HLA-B*27:05 in complex with a SON peptide (RRFSRSPIRR) and its mono- or bi-phosphorylated variants (Table 3). Similarly to the previously described HLA-B*40:02 structure, the authors did not observe significant differences between the crystal structures pSON (RRF(pS) RSPIRR) or SON peptide ligands complexed with HLA-B*27:05. There was also no difference in pMHC binding affinities for these ligands. However, the introduction of a second phosphoserine at P6 resulted in a sharp decrease in binding affinity compared to the SON or pSON peptides. Comparison between the crystal structures for mono (phosphorylation at S4) and bi-phosphorylated (phosphorylation at S4 and S6) peptides revealed that phosphorylation at Ser-P6 causes a conformational switch from an anchor (Ser-P6) to a non-anchor (pSer-P6) position, affecting the hydrogen bonding pattern at the ligand-HLA interface and weakening the binding [73]. Other data suggest that the double-phosphorylated epitopes may exist, but predominantly with a phosphoserine located at P4 and in a penultimate position of an epitope, such as P8 of a 9-mer peptide) [69] (Figure 2C).

However, the relationship between phosphate exposure and epitope immunogenicity remains uncertain, as antigen-specific TCRs were not identified in the above studies. Our recent work [22] provided critical insights into molecular mechanisms underlying the immunogenicity of HLA-B*07:02- specific phosphor-neoantigens by describing the crystal structures for pMHC complexes and a p- peptide-specific TCR (TCR27), revealing the mechanism of p-peptide recognition by the TCR [22]. TCR27 is capable of cross-recognizing two p-peptides detected in acute myeloid leukemia (AML) and melanoma [52,70] – pMLL_747–755_ (EPR(pS)PSHSM) and pDOT1L_998–1006_ (LPA(pS)PAHQL), respectively. We demonstrated that replacing pSer-P4 in pMLL_747–755_ with various phosphomimetics reduced or abolished the interaction with TCR27 [22]. To elucidate the mechanism of p-peptide recognition by the TCR, we solved and compared the crystal structures of HLA-B*07:02 in complex with pMLL_747–755_ (EPR(pS)PSHSM) or pDOT1L_998–1006_ (LPA(pS)PAHQL), with phosphomimetics (phosphono- and sulfo- derivatives of pMLL), or their non-phosphorylated counterparts. Overall, phosphorylation at Ser-P4 (or its modification by phosphomimetics) had little effect on binding affinities between peptide ligands and HLA-B*07:02, which correlated with the lack of significant structural differences between the corresponding pMHC complexes. Structural similarities were found between these and the previously solved p-peptide-MHC structures with other HLA-B molecules, particularly in terms of pSer-P4 orientation, conformation, and high solvent exposure [72,73].

We also determined the crystal structure of TCR27 and used NMR-guided docking to model the ternary complex between HLA-B*07:02, pMLL_747–755_, and TCR27. In this complex, the phosphate moiety plays a crucial role by maintaining an extensive hydrogen bond network with surrounding TCR residues [22] (Figure 2D). This finding explains the sharp reduction in binding between pMHC and TCR27 when pSer-P4 replaced with phosphomimetics. Structural and biophysical analysis of TCR27-pMHC complexes revealed that the phosphate group defines the specificity and strength of TCR binding, which is particularly important for generating robust anti-tumor immune responses, as T cells can more effectively distinguish cancer cells from normal cells. Practically, these data present an opportunity for the rational design of TCRs targeting phospho-neoantigens.

To understand the mechanism of p-peptide binding in the context of MHC Class II and its recognition by CD4^+^ T cells, Li and co-authors [55] determined the crystal structure of pMART1_100–114_ (APPAYEKL(pS)AEQ) in complex with HLA-DRB*01:01 at a 2.1 Å resolution. MART1 is selectively expressed by melanoma and thus serves as a target for cancer vaccines. The crystal structure of the 15-residue pMART_110–114_ revealed Tyr104 (P1) and Ala109 (P6) as primary anchor residues, with a fully solvent-exposed phosphorylated Ser108 (P5). The presence of pSer-P5 slightly reduced the binding affinity of pMART1 to HLA compared to the wild-type peptide but resulted in specific recognition by a CD4^+^ T cell clone, D7-F6. These findings suggest that phosphorylation is a critical determinant of TCR recognition for both MHC I and MHC class II-restricted p-peptides.

## Future Prospects

The cancer immunopeptidome is a treasure trove of tumor-specific and associated peptides that can be utilized in various immunotherapy applications, such as cancer vaccines. Immunopeptides may encompass post-translational modifications that are often overlooked by genomic and transcriptomic tools. These modified peptides are an emerging class of potential targets for shared tumor antigens. While this review focuses on phosphorylation, other modifications like glycosylation, acetylation, and citrullination may also result in cancer-specific modified peptides [76–78].

Phosphorylation may enhance epitope specificity, especially if their expression is restricted to cancer cells. These modifications are not processed in the thymus, allowing T cells to pass through central tolerance. Comprehensive studies on T cell responses to p-peptides, particularly in the context of MHC Class I, underscore the substantial potential for use in cancer therapy and vaccines. A key feature of p-peptides that drives the development of p-peptide-targeted agents is the distinct recognition surface presented by phosphorylated epitope sequences compared to their non-phosphorylated counterparts. Studies have clearly demonstrated that TCRs specific to p-peptides can recognize them without cross-reactivity to the wild-type peptides.

Despite challenges with their identification, phosphorylated antigens remain appealing targets for immunotherapeutic treatments due to their shared expression across different cancer types and potential in TCR therapy as shown by Patskovsky *et al.* [22]. They also hold potential for treating other diseases such as viral infections. Future research could incorporate p-peptides into neoadjuvant settings as cancer vaccines alone or in combination with ICB therapies with a possibility of synergistic effects that could lead to enhanced therapeutic outcomes. Their application as targets for peptide-centric chimeric antigen receptor T cell therapies, as exemplified by recent work by Yamarkovich *et al.* [79] can also be explored. Another important aspect to explore is the intra-tumor and inter-patient heterogeneity in p-peptide presentation and their potential implications for immunotherapy. A more detailed analysis of heterogeneity, both within tumors and across patients would be a valuable area for future investigation and could potentially lead to identification of potential p-peptides and their application in therapies.

In summary, post-translationally modified peptides, such as p-peptides, represent a novel and highly promising target for cancer therapy. The synergy of p-peptides or other post-translationally modified peptides with other forms of immunotherapy could significantly enhance the efficacy and specificity of anti-cancer treatments in the future.

## Figures and Tables

**Table 1.** Protein phosphorylation associated databases and tools.

Databases and tools	Key Features	Refs	URL
**Databases**			
PhosphoSitePlus	Online resource for studying experimentally observed PTMs such as phosphorylation, ubiquitination, and acetylation.	[23]	https://www.phosphosite.org
PHOSIDA	PTMs such as N-glycosylation, phosphorylation, and acetylation of proteins are listed	[24]	http://www.phosida.com
EPSD	Eukaryotic protein phosphorylation sites database	[25]	http://epsd.biocuckoo.cn
Phospho3D	Protein 3-D structures and phosphorylation database	[26]	http://www.phospho3d.org
LymPHOS 2.0	Human T-lymphocyte phosphoproteome database	[27]	https://www.lymphos.org
RegPhos 2.0	Open resource to explore protein kinase-substrate phosphorylation networks in mammals.	[28]	http://140.138.144.141/~RegPhos
PhosphoNET	Provide information on phosphorylation sites in over 20,000 human proteins.	[29]	http://www.phosphonet.ca
BioGRID	Open access PTM database	[30]	https://orcs.thebiogrid.org
**Analysis tools used in MS-based discovery of PTMs**			
MSFragger	Database search tool using fragment ion indexing method to rapidly perform spectra similarity comparisons	[31]	https://msfragger.nesvilab.org/
TagGraph	Tool based on string-based faster search method and probabilistic validation model	[32]	https://sourceforge.net/projects/taggraph/
PTM-Shepherd	Analysis tool of open search results for PTMs and chemical modifications	[33]	https://github.com/Nesvilab/PTM-Shepherd

**Table 2.** Post-translationally modified tumor-associated antigens.

Modification	Source protein	Associated cancer	Immunogenicity	MHC restriction	Refs
Glycosylation	MUC1	Breast cancer	CD8^+^ T cells	Class I	[30]
	Myocyte specific enhancer factor 2C	Leukemia	CD8^+^ T cells	Class I	[21]
	RNA binding protein 27	Leukemia	CD8^+^ T cells	Class I	
	MKL/myocardin-like protein 2	Leukemia	CD8^+^ T cells	Class I	
	E1A-binding protein p400	Leukemia	CD8^+^ T cells	Class I	
Phosphorylation	MART-1	Melanoma	CD4^+^ T cells	Class II	[42]
	IRS2	Melanoma, Breast	CD8^+^ T cells	Class I	[19, 20]
	BCAR3	Melanoma	CD8^+^ T cells	Class I	[20]
	β catenin	Melanoma, Ovarian carcinoma	CD8^+^ T cells	Class I	[20,43]
	Cdc25b	Melanoma, ovarian carcinoma	CD8^+^ T cells	Class I	[43,44]
	TRAP-1	Lung cancer	CD8^+^ T cells	Class I	[42]
	Vimentin	Colorectal cancer	CD4^+^ T Cells	Class II	[45]
	p53	Head and neck SCC	CD4^+^ T Cells	Class II	[46]
Acetylation	p53	Colon, Prostrate	CD4^+^ T Cells	Class II	[47]
	Eno1	Pancreatic ductal carcinoma	CD4^+^ T Cells	Class II	[48,49]

**Table 3.** The crystal structures between distinct HLA molecules, p-peptides, phosphomimeticsand WT non-p-peptides.

PDB ID	HLAtype	Peptide sequence	Protein name	Gene name/Uniprot ID	Main features	Refs
3L6F	HLA-DRB1*01:01	APPAYEKL(pS)AEQ	Melanoma antigen recognized by T cells, MART-1	MLANA/Q16655	Phosphoserine residue at position P5, exposed	[55]
3BH8	HLA-A*02:01	RQA(pS)IELPSM	Lymphocyte specific protein 1	LSP1/P33241	High phosphopeptide affinity	[66]
3BH9	HLA-A*02:01	RTY(pS)GPMNKV	Protein POF1B	POF1B/Q8WVV4	High phosphopeptide affinity	
3BHB	HLA-A*02:01	KMD(pS)FLDMQL	Protein NEDD1	NEDD1/Q8NHV4	High phosphopeptide affinity	
3BGM	HLA-A*02:01	RQA(pS)LSISV	Serine/threonine-protein kinase D2	PRKD2/Q9BZL6	High phosphopeptide affinity	
4NNX	HLA-A*02:01	RQA(pS)LSISV	Serine/threonine-protein kinase D2	PRKD2/Q9BZL6	High phosphopeptide affinity	[67]
4NNY	HLA-A*02:01	RQASLSISV	Serine/threonine-protein kinase D2	PRKD2/Q9BZL6	Low WT peptide affinity	
4NO3	HLA-A*02:01	RQI(pS)QDVKL	AMP deaminase 2	Q01433/AMPD2	High phosphopeptide affinity	
4NO5	HLA-A*02:01	RQISQDVKL	AMP deaminase 2	Q01433/AMPD2	Low WT peptide affinity	
4NO2	HLA-A*02:01	RQA(pS)IELPSMAV	Lymphocyte specific protein 1	LSP1/P33241	Phosphopeptide affinity similar to WT	
4NOo	HLA-A*02:01	RQASIELPSMAV	Lymphocyte specific protein 1	LSP1/P33241	Complex with LILRB1, high affinity WT peptide	
3FQN	HLA-A*02:01	YLDSGIHSGA	Catenin beta-1	CTNNB1/P35222	High peptide affinity	[65]
3FQR	HLA-A*02:01	YLD(pS)GIHSGA	Catenin beta-1	CTNNB1/P35222	Phosphopeptide affinity similar to WT	
3FQT	HLA-A*02:01	GLLGSPVRA	M-phase inducer phosphatase 2	CDC25B/P30305	High peptide affinity	
3FQU	HLA-A*02:01	GLLG(pS)PVRA	M-phase inducer phosphatase 2	CDC25B/P30305	Phosphopeptide affinity similar to WT	
3FQW	HLA-A*02:01	RVASPTSGV	Insulin receptor substrate 2	IRS2/Q9Y4H2	High peptide affinity	
3FQX	HLA-A*02:01	RVA(pS)PTSGV	Insulin receptor substrate 2	IRS2/Q9Y4H2	Phosphopeptide affinity higher than WT	
5IEH	HLA-B*40:02	REF(pS)KEPEL	Inner centromere protein	INCENP/Q9NQS7	Phosphopeptide affinity similar to WT	[72]
5IEK	HLA-B*40:02	REFSKEPEL	Inner centromere protein	INCENP/Q9NQS7	High peptide affinity	
7CIQ	HLA-B*27:05	RRFSRSPIRR	mRNA splicing cofactor, SON	SON/P18583	High peptide affinity	[73]
7CIR	HLA-B*27:05	RRF(pS)RSPIRR	mRNA splicing cofactor, SON	SON/P18583	Peptide affinity similar to WT	
7DYN	HLA-B*27:05	RRF(pS)R(pS)PIR R	mRNA splicing cofactor, SON	SON/P18583	Phosphopeptide affinity much lower than WT	
7CIS	HLA-B*27:05	RRF(pS)R(pS)PIR	mRNA splicing cofactor, SON	SON/P18583	Phosphopeptide affinity much lower than WT	
7RZD	HLA-B*07:02	EPRSPSHSM	Histone-lysine N-methyltransferase 2A	KMT2A/Q03164	High peptide affinity	[22]
7RZJ	HLA-B*07:02	EPR(pS)PSHSM	Histone-lysine N-methyltransferase 2A	KMT2A/Q03164	Phosphopeptide affinity similar to WT	
7S8A	HLA-B*07:02	EPR(pS)PSHSM	Histone-lysine N-methyltransferase 2A	KMT2A/Q03164	Phosphopeptide affinity similar to WT	
7S7D	HLA-B*07:02	EPR(OSE)PSHSM, sulfo-	Histone-lysine N-methyltransfera se 2A	KMT2A/Q03164	Sulfopeptide affinity similar to WT	
7S79	HLA-B*07:02	EPR(E7P)PSHSM, phosphono-	Histone-lysine N-methyltransferase 2A	KMT2A/Q03164	Phosphonopeptide affinity similar to WT	
7S7F	HLA-B*07:02	LPA(pS)PAHQL	Histone-lysine N-methyltransferase, H3 lysine-79 specific	DOT1L/Q8TEK3	Phosphopeptide had slightly lower affinity	
7S7E	HLA-B*07:02	LPASPAHQL	Histone-lysine N-methyltransferase, H3 lysine-79 specific	DOT1L/Q8TEK3	High peptide affinity	

## References

[1] Hodi FS, O’Day SJ, McDermott DF, Weber RW, Sosman JA, Haanen JB, Improved survival with ipilimumab in patients with metastatic melanoma. N Engl J Med. 2010 Aug 19;363(8):711–23. 20525992 10.1056/NEJMoa1003466 PMC3549297

[2] Larkin J, Chiarion-Sileni V, Gonzalez R, Grob JJ, Cowey CL, Lao CD, Combined Nivolumab and Ipilimumab or Monotherapy in Untreated Melanoma. N Engl J Med. 2015 Jul 2;373(1):23–34. 26027431 10.1056/NEJMoa1504030 PMC5698905

[3] Robert C, Schachter J, Long GV, Arance A, Grob JJ, Mortier L, Pembrolizumab versus Ipilimumab in Advanced Melanoma. N Engl J Med. 2015 Jun 25;372(26):2521–32. 25891173 10.1056/NEJMoa1503093

[4] Mazieres J, Drilon A, Lusque A, Mhanna L, Cortot AB, Mezquita L, Immune checkpoint inhibitors for patients with advanced lung cancer and oncogenic driver alterations: results from the IMMUNOTARGET registry. Ann Oncol. 2019 Aug 1;30(8):1321–8. 31125062 10.1093/annonc/mdz167 PMC7389252

[5] Yang C, Xia BR, Zhang ZC, Zhang YJ, Lou G, Jin WL. Immunotherapy for Ovarian Cancer: Adjuvant, Combination, and Neoadjuvant. Front Immunol. 2020 Oct 6;11:577869. 33123161 10.3389/fimmu.2020.577869 PMC7572849

[6] Aggarwal C, Ben-Shachar R, Gao Y, Hyun SW, Rivers Z, Epstein C, Assessment of Tumor Mutational Burden and Outcomes in Patients With Diverse Advanced Cancers Treated With Immunotherapy. JAMA Netw Open. 2023 May 1;6(5):e2311181. 37129893 10.1001/jamanetworkopen.2023.11181 PMC10155064

[7] Hotz MJ, O’Halloran EA, Hill MV, Hayden K, Zaladonis AG, Deng M, Tumor mutational burden and somatic mutation status to predict disease recurrence in advanced melanoma. Melanoma Res. 2022 Apr 1;32(2):112–9. 35213415 10.1097/CMR.0000000000000808 PMC9109603

[8] Xie N, Shen G, Gao W, Huang Z, Huang C, Fu L. Neoantigens: promising targets for cancer therapy. Signal Transduct Target Ther. 2023 Jan 6;8(1):9. 36604431 10.1038/s41392-022-01270-x PMC9816309

[9] Zheng M Tumor mutation burden for predicting immune checkpoint blockade response: the more, the better. J Immunother Cancer. 2022 Jan;10(1):e003087. 35101940 10.1136/jitc-2021-003087 PMC8804687

[10] Huang AC, Zappasodi R. A decade of checkpoint blockade immunotherapy in melanoma: understanding the molecular basis for immune sensitivity and resistance. Nat Immunol. 2022 May;23(5):660–70. 35241833 10.1038/s41590-022-01141-1 PMC9106900

[11] Vlad AM, Muller S, Cudic M, Paulsen H, Otvos L Jr, Hanisch FG, Complex carbohydrates are not removed during processing of glycoproteins by dendritic cells: processing of tumor antigen MUC1 glycopeptides for presentation to major histocompatibility complex class II-restricted T cells. J Exp Med. 2002 Dec 2;196(11):1435–46. 12461079 10.1084/jem.20020493 PMC2194269

[12] Apostolopoulos V, Yuriev E, Ramsland PA, Halton J, Osinski C, Li W, A glycopeptide in complex with MHC class I uses the GalNAc residue as an anchor. Proc Natl Acad Sci U S A. 2003 Dec 9;100(25):15029–34. 14657390 10.1073/pnas.2432220100 PMC299892

[13] Wang E, Aifantis I. RNA Splicing and Cancer. Trends Cancer. 2020 Aug;6(8):631–44. 32434734 10.1016/j.trecan.2020.04.011

[14] Hoyos LE, Abdel-Wahab O. Cancer-Specific Splicing Changes and the Potential for Splicing-Derived Neoantigens. Cancer Cell. 2018 Aug 13;34(2):181–3. 30107172 10.1016/j.ccell.2018.07.008 PMC6614861

[15] Kahles A, Lehmann KV, Toussaint NC, Hüser M, Stark SG, Sachsenberg T, Comprehensive Analysis of Alternative Splicing Across Tumors from 8,705 Patients. Cancer Cell. 2018 Aug 13;34(2):211–24.e6. 30078747 10.1016/j.ccell.2018.07.001 PMC9844097

[16] Marijt KA, Doorduijn EM, van Hall T. TEIPP antigens for T-cell based immunotherapy of immune-edited HLA class Ilow cancers. Mol Immunol. 2019 Sep;113:43–9. 29627136 10.1016/j.molimm.2018.03.029

[17] van Hall T, Wolpert EZ, van Veelen P, Laban S, van der Veer M, Roseboom M, Selective cytotoxic T-lymphocyte targeting of tumor immune escape variants. Nat Med. 2006 Apr;12(4):417–24. 16550190 10.1038/nm1381

[18] RodrÍguez E, Schetters STT, van Kooyk Y. The tumour glyco-code as a novel immune checkpoint for immunotherapy. Nat Rev Immunol. 2018 Mar;18(3):204–11. 29398707 10.1038/nri.2018.3

[19] Dao T, Mun SS, Molvi Z, Korontsvit T, Klatt MG, Khan AG, A TCR mimic monoclonal antibody reactive with the “public” phospho-neoantigen pIRS2/HLA-A*02:01 complex. JCI Insight. 2022 Mar 8;7(5):e151624. 35260532 10.1172/jci.insight.151624 PMC8983142

[20] Engelhard VH, Obeng RC, Cummings KL, Petroni GR, Ambakhutwala AL, Chianese-Bullock KA, MHC-restricted phosphopeptide antigens: preclinical validation and first-in-humans clinical trial in participants with high-risk melanoma. J Immunother Cancer. 2020 May;8(1):e000262. 32385144 10.1136/jitc-2019-000262 PMC7228659

[21] Malaker SA, Penny SA, Steadman LG, Myers PT, Loke JC, Raghavan M, Identification of Glycopeptides as Posttranslationally Modified Neoantigens in Leukemia. Cancer Immunol Res. 2017 May;5(5):376–84. 28314751 10.1158/2326-6066.CIR-16-0280 PMC5508727

[22] Patskovsky Y, Natarajan A, Patskovska L, Nyovanie S, Joshi B, Morin B, Molecular mechanism of phosphopeptide neoantigen immunogenicity. Nat Commun. 2023 Jun 23;14(1):3763. 37353482 10.1038/s41467-023-39425-1 PMC10290117

[23] Hornbeck PV, Zhang B, Murray B, Kornhauser JM, Latham V, Skrzypek E. PhosphoSitePlus, 2014: mutations, PTMs and recalibrations. Nucleic Acids Res. 2015 Jan;43(Database issue):D512–20. 25514926 10.1093/nar/gku1267 PMC4383998

[24] Gnad F, Gunawardena J, Mann M. PHOSIDA 2011: the posttranslational modification database. Nucleic Acids Res. 2011 Jan;39(Database issue):D253–60. 21081558 10.1093/nar/gkq1159 PMC3013726

[25] Lin S, Wang C, Zhou J, Shi Y, Ruan C, Tu Y, EPSD: a well-annotated data resource of protein phosphorylation sites in eukaryotes. Brief Bioinform. 2021 Jan 18;22(1):298–307. 32008039 10.1093/bib/bbz169

[26] Zanzoni A, Ausiello G, Via A, Gherardini PF, Helmer-Citterich M. Phospho3D: a database of three-dimensional structures of protein phosphorylation sites. Nucleic Acids Res. 2007 Jan;35(Database issue):D229–31. 17142231 10.1093/nar/gkl922 PMC1669737

[27] Nguyen TD, Vidal-Cortes O, Gallardo O, Abian J, Carrascal M. LymPHOS 2.0: an update of a phosphosite database of primary human T cells. Database (Oxford). 2015 Dec 26;2015:bav115. 26708986 10.1093/database/bav115 PMC4691341

[28] Huang KY, Wu HY, Chen YJ, Lu CT, Su MG, Hsieh YC, RegPhos 2.0: an updated resource to explore protein kinase-substrate phosphorylation networks in mammals. Database (Oxford). 2014 Apr 25;2014(0):bau034. 24771658 10.1093/database/bau034 PMC3999940

[29] Safaei J, Maňuch J, Gupta A, Stacho L, Pelech S. Prediction of 492 human protein kinase substrate specificities. Proteome Sci. 2011 Oct 14;9 Suppl 1(Suppl 1):S6. 22165948 10.1186/1477-5956-9-S1-S6 PMC3379035

[30] Oughtred R, Stark C, Breitkreutz BJ, Rust J, Boucher L, Chang C, The BioGRID interaction database: 2019 update. Nucleic Acids Res. 2019 Jan 8;47(D1):D529–41. 30476227 10.1093/nar/gky1079 PMC6324058

[31] Kong AT, Leprevost FV, Avtonomov DM, Mellacheruvu D, Nesvizhskii AI. MSFragger: ultrafast and comprehensive peptide identification in mass spectrometry-based proteomics. Nat Methods. 2017 May;14(5):513–20. 28394336 10.1038/nmeth.4256 PMC5409104

[32] Devabhaktuni A, Lin S, Zhang L, Swaminathan K, Gonzalez CG, Olsson N, TagGraph reveals vast protein modification landscapes from large tandem mass spectrometry datasets. Nat Biotechnol. 2019 Apr;37(4):469–79. 30936560 10.1038/s41587-019-0067-5 PMC6447449

[33] Geiszler DJ, Kong AT, Avtonomov DM, Yu F, Leprevost FDV, Nesvizhskii AI. PTM-Shepherd: Analysis and Summarization of Post-Translational and Chemical Modifications From Open Search Results. Mol Cell Proteomics. 2021;20:100018. 33568339 10.1074/mcp.TIR120.002216 PMC7950090

[34] Turdo A, D’Accardo C, Glaviano A, Porcelli G, Colarossi C, Colarossi L, Targeting Phosphatases and Kinases: How to Checkmate Cancer. Front Cell Dev Biol. 2021 Oct 28;9:690306. 34778245 10.3389/fcell.2021.690306 PMC8581442

[35] Vainonen JP, Momeny M, Westermarck J. Druggable cancer phosphatases. Sci Transl Med. 2021 Apr 7;13(588):eabe2967. 33827975 10.1126/scitranslmed.abe2967

[36] Sangodkar J, Farrington CC, McClinch K, Galsky MD, Kastrinsky DB, Narla G. All roads lead to PP2A: exploiting the therapeutic potential of this phosphatase. FEBS J. 2016 Mar;283(6):1004–24. 26507691 10.1111/febs.13573 PMC4803620

[37] Ruvolo PP. The broken “Off” switch in cancer signaling: PP2A as a regulator of tumorigenesis, drug resistance, and immune surveillance. BBA Clin. 2016 Aug 3;6:87–99. 27556014 10.1016/j.bbacli.2016.08.002 PMC4986044

[38] Mahoney KE, Shabanowitz J, Hunt DF. MHC Phosphopeptides: Promising Targets for Immunotherapy of Cancer and Other Chronic Diseases. Mol Cell Proteomics. 2021;20:100112. 34129940 10.1016/j.mcpro.2021.100112 PMC8724925

[39] Lucas CM, Harris RJ, Holcroft AK, Scott LJ, Carmell N, McDonald E, Second generation tyrosine kinase inhibitors prevent disease progression in high-risk (high CIP2A) chronic myeloid leukaemia patients. Leukemia. 2015 Jul;29(7):1514–23. 25765543 10.1038/leu.2015.71

[40] Vélez-Cruz R, Johnson DG. The Retinoblastoma (RB) Tumor Suppressor: Pushing Back against Genome Instability on Multiple Fronts. Int J Mol Sci. 2017 Aug 16;18(8):1776. 28812991 10.3390/ijms18081776 PMC5578165

[41] Kastenhuber ER, Lowe SW. Putting p53 in Context. Cell. 2017 Sep 7;170(6):1062–78. 28886379 10.1016/j.cell.2017.08.028 PMC5743327

[42] Lin MH, Shen KY, Liu BS, Chen IH, Sher YP, Tseng GC, Immunological evaluation of a novel HLA-A2 restricted phosphopeptide of tumor associated Antigen, TRAP1, on cancer therapy. Vaccine X. 2019 Mar 11;1:100017. 31384738 10.1016/j.jvacx.2019.100017 PMC6668235

[43] Zarling AL, Polefrone JM, Evans AM, Mikesh LM, Shabanowitz J, Lewis ST, Identification of class I MHC-associated phosphopeptides as targets for cancer immunotherapy. Proc Natl Acad Sci U S A. 2006 Oct 3;103(40):14889–94. 17001009 10.1073/pnas.0604045103 PMC1595446

[44] Zarling AL, Obeng RC, Desch AN, Pinczewski J, Cummings KL, Deacon DH, MHC-restricted phosphopeptides from insulin receptor substrate-2 and CDC25b offer broad-based immunotherapeutic agents for cancer. Cancer Res. 2014 Dec 1;74(23):6784–95. 25297629 10.1158/0008-5472.CAN-14-0043 PMC4252710

[45] Ohara M, Ohara K, Kumai T, Ohkuri T, Nagato T, Hirata-Nozaki Y, Phosphorylated vimentin as an immunotherapeutic target against metastatic colorectal cancer. Cancer Immunol Immunother. 2020 Jun;69(6):989–99. 32086539 10.1007/s00262-020-02524-9 PMC11027720

[46] Ohara K, Ohkuri T, Kumai T, Nagato T, Nozaki Y, Ishibashi K, Targeting phosphorylated p53 to elicit tumor-reactive T helper responses against head and neck squamous cell carcinoma. Oncoimmunology. 2018 Aug 1;7(9):e1466771. 30510853 10.1080/2162402X.2018.1466771 PMC6259824

[47] Kumai T, Ishibashi K, Oikawa K, Matsuda Y, Aoki N, Kimura S, Induction of tumor-reactive T helper responses by a posttranslational modified epitope from tumor protein p53. Cancer Immunol Immunother. 2014 May;63(5):469–78. 24633296 10.1007/s00262-014-1533-z PMC11028558

[48] Zhou W, Capello M, Fredolini C, Piemonti L, Liotta LA, Novelli F, Mass spectrometry analysis of the post-translational modifications of alpha-enolase from pancreatic ductal adenocarcinoma cells. J Proteome Res. 2010 Jun 4;9(6):2929–36. 20433201 10.1021/pr901109w

[49] Capello M, Caorsi C, Bogantes Hernandez PJ, Dametto E, Bertinetto FE, Magistroni P, Phosphorylated alpha-enolase induces autoantibodies in HLA-DR8 pancreatic cancer patients and triggers HLA-DR8 restricted T-cell activation. Immunol Lett. 2015 Sep;167(1):11–6. 26096821 10.1016/j.imlet.2015.06.008

[50] Wu JQ, Guo JY, Tang W, Yang CS, Freel CD, Chen C, PP1-mediated dephosphorylation of phosphoproteins at mitotic exit is controlled by inhibitor-1 and PP1 phosphorylation. Nat Cell Biol. 2009 May;11(5):644–51. 19396163 10.1038/ncb1871 PMC2788612

[51] Jurtz V, Paul S, Andreatta M, Marcatili P, Peters B, Nielsen M. NetMHCpan-4.0: Improved Peptide-MHC Class I Interaction Predictions Integrating Eluted Ligand and Peptide Binding Affinity Data. J Immunol. 2017 Nov 1;199(9):3360–8. 28978689 10.4049/jimmunol.1700893 PMC5679736

[52] Bassani-Sternberg M, Bräunlein E, Klar R, Engleitner T, Sinitcyn P, Audehm S, Direct identification of clinically relevant neoepitopes presented on native human melanoma tissue by mass spectrometry. Nat Commun. 2016 Nov 21;7:13404. 27869121 10.1038/ncomms13404 PMC5121339

[53] Zarling AL, Ficarro SB, White FM, Shabanowitz J, Hunt DF, Engelhard VH. Phosphorylated peptides are naturally processed and presented by major histocompatibility complex class I molecules in vivo. J Exp Med. 2000 Dec 18;192(12):1755–62. 11120772 10.1084/jem.192.12.1755 PMC2213507

[54] Penny SA, Abelin JG, Malaker SA, Myers PT, Saeed AZ, Steadman LG, Tumor Infiltrating Lymphocytes Target HLA-I Phosphopeptides Derived From Cancer Signaling in Colorectal Cancer. Front Immunol. 2021 Aug 24;12:723566. 34504498 10.3389/fimmu.2021.723566 PMC8421858

[55] Li Y, Depontieu FR, Sidney J, Salay TM, Engelhard VH, Hunt DF, Structural basis for the presentation of tumor-associated MHC class II-restricted phosphopeptides to CD4+ T cells. J Mol Biol. 2010 Jun 18;399(4):596–603. 20417641 10.1016/j.jmb.2010.04.037 PMC2904831

[56] Depontieu FR, Qian J, Zarling AL, McMiller TL, Salay TM, Norris A, Identification of tumor-associated, MHC class II-restricted phosphopeptides as targets for immunotherapy. Proc Natl Acad Sci U S A. 2009 Jul 21;106(29):12073–8. 19581576 10.1073/pnas.0903852106 PMC2715484

[57] Meyer VS, Drews O, Günder M, Hennenlotter J, Rammensee HG, Stevanovic S. Identification of natural MHC class II presented phosphopeptides and tumor-derived MHC class I phospholigands. J Proteome Res. 2009 Jul;8(7):3666–74. 19415920 10.1021/pr800937k

[58] Schunter AJ, Yue X, Hummon AB. Phosphoproteomics of colon cancer metastasis: comparative mass spectrometric analysis of the isogenic primary and metastatic cell lines SW480 and SW620. Anal Bioanal Chem. 2017 Mar;409(7):1749–63. 27987026 10.1007/s00216-016-0125-5 PMC5303640

[59] Aikio E, Koivukoski S, Kallio E, Sadeesh N, Niskanen EA, Latonen L. Complementary analysis of proteome-wide proteomics reveals changes in RNA binding protein-profiles during prostate cancer progression. Cancer Rep (Hoboken). 2023 Oct;6(10):e1886. 37591798 10.1002/cnr2.1886 PMC10598248

[60] Drake JM, Paull EO, Graham NA, Lee JK, Smith BA, Titz B, Phosphoproteome Integration Reveals Patient-Specific Networks in Prostate Cancer. Cell. 2016 Aug 11;166(4):1041–54. 27499020 10.1016/j.cell.2016.07.007 PMC4985183

[61] Lin X, Tang S, Guo Y, Tang R, Li Z, Pan X, Personalized neoantigen vaccine enhances the therapeutic efficacy of bevacizumab and anti-PD-1 antibody in advanced non-small cell lung cancer. Cancer Immunol Immunother. 2024 Jan 27;73(2):26. 38280084 10.1007/s00262-023-03598-x PMC10821847

[62] Imani S, Tagit O, Pichon C. Neoantigen vaccine nanoformulations based on Chemically synthesized minimal mRNA (CmRNA): small molecules, big impact. NPJ Vaccines. 2024 Jan 18;9(1):14. 38238340 10.1038/s41541-024-00807-1 PMC10796345

[63] Li J, Xiao Z, Wang D, Jia L, Nie S, Zeng X, The screening, identification, design and clinical application of tumor-specific neoantigens for TCR-T cells. Mol Cancer. 2023 Aug 30;22(1):141. 37649123 10.1186/s12943-023-01844-5 PMC10466891

[64] Palmer CD, Rappaport AR, Davis MJ, Hart MG, Scallan CD, Hong SJ, Individualized, heterologous chimpanzee adenovirus and self-amplifying mRNA neoantigen vaccine for advanced metastatic solid tumors: phase 1 trial interim results. Nat Med. 2022 Aug;28(8):1619–29. 35970920 10.1038/s41591-022-01937-6

[65] Petersen J, Wurzbacher SJ, Williamson NA, Ramarathinam SH, Reid HH, Nair AK, Phosphorylated self-peptides alter human leukocyte antigen class I-restricted antigen presentation and generate tumor-specific epitopes. Proc Natl Acad Sci U S A. 2009 Feb 24;106(8):2776–81. 19196958 10.1073/pnas.0812901106 PMC2650342

[66] Mohammed F, Cobbold M, Zarling AL, Salim M, Barrett-Wilt GA, Shabanowitz J, Phosphorylation-dependent interaction between antigenic peptides and MHC class I: a molecular basis for the presentation of transformed self. Nat Immunol. 2008 Nov;9(11):1236–43. 18836451 10.1038/ni.1660 PMC2596764

[67] Mohammed F, Stones DH, Zarling AL, Willcox CR, Shabanowitz J, Cummings KL, The antigenic identity of human class I MHC phosphopeptides is critically dependent upon phosphorylation status. Oncotarget. 2017 Apr 8;8(33):54160–72. 28903331 10.18632/oncotarget.16952 PMC5589570

[68] Amanchy R, Periaswamy B, Mathivanan S, Reddy R, Tattikota SG, Pandey A. A curated compendium of phosphorylation motifs. Nat Biotechnol. 2007 Mar;25(3):285–6. 17344875 10.1038/nbt0307-285

[69] Solleder M, Guillaume P, Racle J, Michaux J, Pak HS, Müller M, Mass Spectrometry Based Immunopeptidomics Leads to Robust Predictions of Phosphorylated HLA Class I Ligands. Mol Cell Proteomics. 2020 Feb;19(2):390–404. 31848261 10.1074/mcp.TIR119.001641 PMC7000122

[70] Cobbold M, De La Peña H, Norris A, Polefrone JM, Qian J, English AM, MHC class I-associated phosphopeptides are the targets of memory-like immunity in leukemia. Sci Transl Med. 2013 Sep 18;5(203):203ra125. 10.1126/scitranslmed.3006061 PMC4071620 24048523

[71] Marcilla M, Alpízar A, Lombardía M, Ramos-Fernandez A, Ramos M, Albar JP. Increased diversity of the HLA-B40 ligandome by the presentation of peptides phosphorylated at their main anchor residue. Mol Cell Proteomics. 2014 Feb;13(2):462–74. 24366607 10.1074/mcp.M113.034314 PMC3916647

[72] Alpízar A, Marino F, Ramos-Fernández A, Lombardía M, Jeko A, Pazos F, A Molecular Basis for the Presentation of Phosphorylated Peptides by HLA-B Antigens. Mol Cell Proteomics. 2017 Feb;16(2):181–93. 27920218 10.1074/mcp.M116.063800 PMC5294207

[73] Zhao Y, Sun M, Zhang N, Liu X, Yue C, Feng L, Phosphosite-dependent presentation of dual phosphorylated peptides by MHC class I molecules. iScience. 2022 Mar 1;25(4):104013. 35310951 10.1016/j.isci.2022.104013 PMC8931367

[74] Liu C, Li Y, Semenov M, Han C, Baeg GH, Tan Y, Control of beta-catenin phosphorylation/degradation by a dual-kinase mechanism. Cell. 2002 Mar 22;108(6):837–47. 11955436 10.1016/s0092-8674(02)00685-2

[75] Orford K, Crockett C, Jensen JP, Weissman AM, Byers SW. Serine phosphorylation-regulated ubiquitination and degradation of beta-catenin. J Biol Chem. 1997 Oct 3;272(40):24735–8. 9312064 10.1074/jbc.272.40.24735

[76] Katayama H, Kobayashi M, Irajizad E, Sevillarno A, Patel N, Mao X, Protein citrullination as a source of cancer neoantigens. J Immunother Cancer. 2021 Jun;9(6):e002549. 34112737 10.1136/jitc-2021-002549 PMC8194337

[77] Pinho SS, Reis CA. Glycosylation in cancer: mechanisms and clinical implications. Nat Rev Cancer. 2015 Sep;15(9):540–55. 26289314 10.1038/nrc3982

[78] Zhong Q, Xiao X, Qiu Y, Xu Z, Chen C, Chong B, Protein posttranslational modifications in health and diseases: Functions, regulatory mechanisms, and therapeutic implications. MedComm (2020). 2023 May 2;4(3):e261. 37143582 10.1002/mco2.261 PMC10152985

[79] Yarmarkovich M, Marshall QF, Warrington JM, Premaratne R, Farrel A, Groff D, Targeting of intracellular oncoproteins with peptide-centric CARs. Nature. 2023 Nov;623(7988):820–27. 37938771 10.1038/s41586-023-06706-0 PMC10665195

